# An Anomalous Case of Horny Excrescences

**Published:** 1851-05

**Authors:** Asbury Evans

**Affiliations:** Covington, Ky.


### THE WESTERN JOURNAL OF MEDICINE AND SURGERY.

MAY, 1851.

Art. I.—*An Anomalous Case of Horny Excrescences.* By Asbury Evans, M. D., of Covington, Ky.

Mrs B——, of Grant county, called on me about the first of February last for treatment of a singular affection of the nails and fingers of both hands.

Her general appearance was that of feebleness, but not positive ill health. She had been married several years, and is the mother of three healthy children. Six months before she presented herself at my office, and immediately after an attack of epidemic dysentery, she had first begun to be affected by the disease. The affection had made great progress in some of the fingers, while in others it had just begun: that which was most diseased was the right thumb; the least so was the left little finger.

The disease began by the deposit of a horny layer beneath the fine extremity of the nail, and then extended inwards, separating the cutis and nail. Successive layers were added, until a mass was produced; having the shape of a wedge with the base looking towards the end of the finger. Upon the further growth of this excrescence, its base would raise up the end of the nail while its edge would continue to insert underneath the length of the nail. The presence of this foreign matter and the upturning of the nails would in no great while produce inflammation of the cutis upon which the horny substance laid, and along the edges and at the roots of the nails, and finally ulceration would result, particularly at the roots of the nails. Eversion of half the length of the nail of the right thumb was produced, and the cutis from which it had been removed was inflamed and deeply ulcerated—the whole exhibiting a most unseemly appearance. Such was the soreness of many of the fingers that, in addition to the pain and uncomfortableness of her situation, Mrs. B. was wholly deprived of the use of her hands.

She had been under the treatment of two physicians, and had, besides, gone the usual round in the employment of salves, ointments and teas so liberally prescribed by the male and female Lady Bountifuls, indigenous alike to country neighborhoods and wealthy and fashionable cities, without improvement, except for a short time, when suffering from a slight ptyalism.

The horny excrescences, seemed to me, to result from an abnormal action of the cutis upon which the nails rest for support, and which had assumed the office of secreting nail matter that, in health, belongs solely to the matrix.

The inflammatory and ulcerative processes were the mechanical consequences of the eversions of the nails, produced by the wedges of horny matter thrown off from the cutis. To subdue this unnatural secretory process of the subungual cutis, was what appeared to be demanded for a cure.

The utility of gentle mercurialization in *Onychia Maligna*, which the ulcers she had, much resembled, and the fact that when slightly salivated she had been benefited, determined me to put her upon the use of mercury. I accordingly prescribed pills of blue mass and opium, daily until her mouth was sore; and applied Fowler's Solution three times a day to the inflamed and ulcerated parts. In ten days her mouth was sore, and the fingers presented a greatly improved appearance. I then gave her large doses of the hydriodate of potash in the compound decoction of sarsaparilla.

I saw her a few days since. Her nails had grown out, and although a little warped, were healthy; the inflammation had disappeared and she had as good use of her hands as before the attack.

March 23d, 1851.

